# Ferroptosis of Macrophages and Endothelial Cells in Atherosclerosis: Molecular Mechanisms and Therapeutic Targets

**DOI:** 10.31083/RCM45117

**Published:** 2025-10-29

**Authors:** Meiling Jiang, Xu Xu, Guofu Zhu

**Affiliations:** ^1^Cardiology Department, The Second Affiliated Hospital of Kunming Medical University, 650000 Kunming, Yunnan, China; ^2^General Medicine Department, Affiliated Calmette Hospital of Kunming Medical University, 650000 Kunming, Yunnan, China

**Keywords:** ferroptosis, atherosclerosis, macrophages, endothelial cells, molecular mechanisms, therapeutic targets

## Abstract

Atherosclerosis (AS) is a significant contributor to cardiovascular disease, characterized by abnormal lipid metabolism, cellular apoptosis, oxidative stress, and chronic inflammation. Ferroptosis represents a form of non-apoptotic programmed cell death characterized by the iron-dependent accumulation of lethal lipid reactive oxygen species (ROS) and the peroxidation of membrane polyunsaturated fatty acid phospholipids (PUFA-PLs). The ferroptosis of endothelial cells (ECs) and macrophages plays a crucial role in the development of atherosclerotic plaques. This review summarizes the mechanisms and associated therapeutic targets related to ferroptosis in macrophages and ECs within the context of AS. Recent research has made substantial progress in elucidating the mechanisms through which ferroptosis influences AS progression; however, a comprehensive understanding of the precise molecular basis for AS remains essential. Moreover, further clinical trials of drugs targeting ferroptosis are necessary. This review updates the knowledge of ferroptosis in ECs and macrophages related to AS, identifies potential links and the subsequent implications for plaque stability, and serves as a reference for developing new pharmacological strategies to address AS and stabilize vulnerable plaques.

## 1. Introduction

Atherosclerosis (AS) significantly contributes to the morbidity and mortality of cardiovascular diseases, accounting for 31% of global deaths [1]. AS is a progressive disease caused by the buildup of low-density lipoprotein (LDL) in the subendothelial matrix. It is marked by endothelial damage, inflammatory cell infiltration, cellular proliferation, and lipid deposition. The onset and progression of AS, as well as plaque rupture, are closely linked to the damage inflicted on vascular cells, including endothelial cells (ECs), smooth muscle cells (SMCs), and macrophages [2]. Notably, the involvement of ferroptosis in the initiation, progression, and pathogenesis of AS and its complications has been increasingly elucidated. Lipid oxidation, excessive cell death, and iron deposition are prominent characteristics observed in human atherosclerotic plaques [3]. Iron exacerbates endothelial dysfunction while promoting smooth muscle cell calcification and facilitating foam cell formation related to macrophages through mechanisms involving oxidative stress, inflammation, and ferroptosis. Additionally, iron overload accelerates ferroptosis, contributing to plaque instability and disease progression [4].

Ferroptosis is a regulated form of cell death marked by the accumulation of reactive oxygen species (ROS) due to abnormal iron metabolism, lipid peroxidation, and disrupted amino acid metabolism [5]. It plays a crucial role in three key stages of AS: endothelial cell injury, monocyte adhesion, and foam cell formation [6]. Ferroptosis damages ECs, impairing their barrier function and allowing LDL particles to infiltrate the subendothelial space where they oxidize into oxidized LDL (ox-LDL). Macrophages then phagocytize ox-LDL, transforming into foam cells—a hallmark of atherosclerotic lesions [7]. Ferroptosis occurs due to a oxidation-reduction (REDOX) imbalance between oxidant and antioxidant production, regulated by an integrated oxidative-antioxidant system [8, 9]. In advanced plaques, up to 50% of dead cells are macrophages; their death is significant for plaque instability and necrotic core formation [2]. Ferroptosis in both endothelial and foam cells contributes to plaque progression and instability. Treatment with ferroptosis inhibitors has been shown to reduce ferroptosis development as well as hyperlipidemia and AS lesions [6]. Ferroptosis inhibitors (ferrostatin-1 or liproxstatin-1) mitigate ferroptosis by decreasing monocyte adhesion and enhancing cholesterol efflux, thereby reducing foam cell formation. Ferroptosis inhibitors primarily consist of iron chelators and lipophilic reactive thiol antioxidants (RTAs) [10]. The study by Yang *et al*. [6] highlights the critical role of ox-LDL in AS pathogenesis, notably promoting ferroptosis in ECs. Ferroptosis is crucial for foam cell formation and lipid accumulation by regulating cholesterol efflux from macrophages. Research indicates that ox-LDL induces ferroptosis by inhibiting glutathione peroxidase 4 (GPX4), a key enzyme that scavenges lipid peroxides.

Anti-ferroptosis therapy shows promise *in vivo*, and ferroptosis-related indicators may aid in diagnosing AS patients. Targeting ferroptosis in ECs and macrophages could provide new strategies for AS treatment. Unlike previous reviews, we focus on ECs and macrophages. From the perspective of ferroptosis, we summarize the mechanisms underlying ferroptosis in macrophages and endothelial cells as they relate to AS. Additionally, we highlight recent advancements in targeted ferroptosis therapies for AS, aiming to provide a theoretical foundation for future novel treatments for AS.

## 2. Ferroptosis in Atherosclerosis

### 2.1 Molecular Mechanisms of Ferroptosis

Iron (Fe) is a vital micronutrient for the human body, essential for oxygen transport, mitochondrial respiration, and REDOX reactions. Under normal conditions, duodenal epithelial cells absorb dietary iron, macrophages reclaim hemoglobin iron from aging red blood cells, and hepatocytes store excess iron. Iron homeostasis relies on a balance between absorption, exportation, utilization, and storage [5]. Cytosolic iron in enterocytes can be stored as ferritin or exported to plasma via ferroportin (FPN). It binds to transferrin (TF) for cellular transport. Before crossing the cell membrane, ferroreductase Cybrd1 (DcytB) reduces non-heme trivalent iron (Fe^3+^) to ferrous iron (Fe^2+^), which is then absorbed by divalent metal transporter 1 (DMT1). This process allows ferrous iron uptake into intestinal cells for distribution according to specific cellular needs [11]. The human body contains about 2–5 grams of total iron; most is bound within heme or other proteins in hemoglobin and myoglobin. Only around 0.1% exists extracellularly—mainly bound to transferrin in serum. Proper maintenance of iron homeostasis is crucial for normal physiological functions across various systems. Excessive intracellular Fe^2+^ accumulation can lead to lipid peroxidation through Fenton reactions and result in ferroptosis [12].

The concept of cell death has expanded beyond apoptosis and necrosis to include additional forms such as necroptosis and ferroptosis [13]. Ferroptosis is a novel form of iron-dependent regulated cell death (RCD) characterized by the accumulation and REDOX imbalance of lipid peroxides. It exhibits distinct morphological, biochemical, and genetic features (Table 1) [14, 15].

**Table 1.** **Distinctions between ferroptosis and other forms of regulated cell death**.

Characteristics (Categories)	Morphological characteristics	Biochemical characteristics	Immunological characteristics	Key proteins
Ferroptosis	Mitochondrial volume decreased, bilayer membrane density increased, mitochondrial cristae were reduced or absent, and the outer mitochondrial membrane showed signs of rupture.	Iron accumulates and lipid peroxidation occurs.	Release DAMPs, pro-inflammatory.	GPX4, TFR1, ferritin, SLC7A11, NRF2, p53, ACSL4, FSP1
Apoptosis	Cell and nuclear volumes decreased, chromatin condensed, nuclei fragmented, apoptotic bodies formed, and the cytoskeleton disintegrated.	Caspase activation and DNA fragmentation.	It generally does not provoke an inflammatory response.	Caspase, Bcl-2, Bax, p53, Fas
Necrotizing Apoptosis	Cells and organelles showed swelling, chromatin was moderately condensed, membranes were compromised, and cellular components were released.	ATP levels decreased, along with the activation of RIP1, RIP3, and MLKL.	Usually release DAMPs, proinflammatory.	RIP1, RIP3

SLC7A11, solute carrier family 7 member 11; GPX4, glutathione peroxidase 4; TFR1, transferrin receptor 1; NRF2, nuclear factor erythroid-2-related factor 2; ACSL4, acyl-CoA synthetase long-chain family member 4; FSP1, ferroptosis suppressor protein 1; Bcl-2, B-cell lymphoma-2; RIP1, receptor-interacting protein 1; RIP3, receptor-interacting protein 3; ATP, adenosine triphosphate; MLKL, mixed lineage kinase-like; DAMPs, damage-associated molecular patterns; p53, protein 53.

**Fig. 1.**
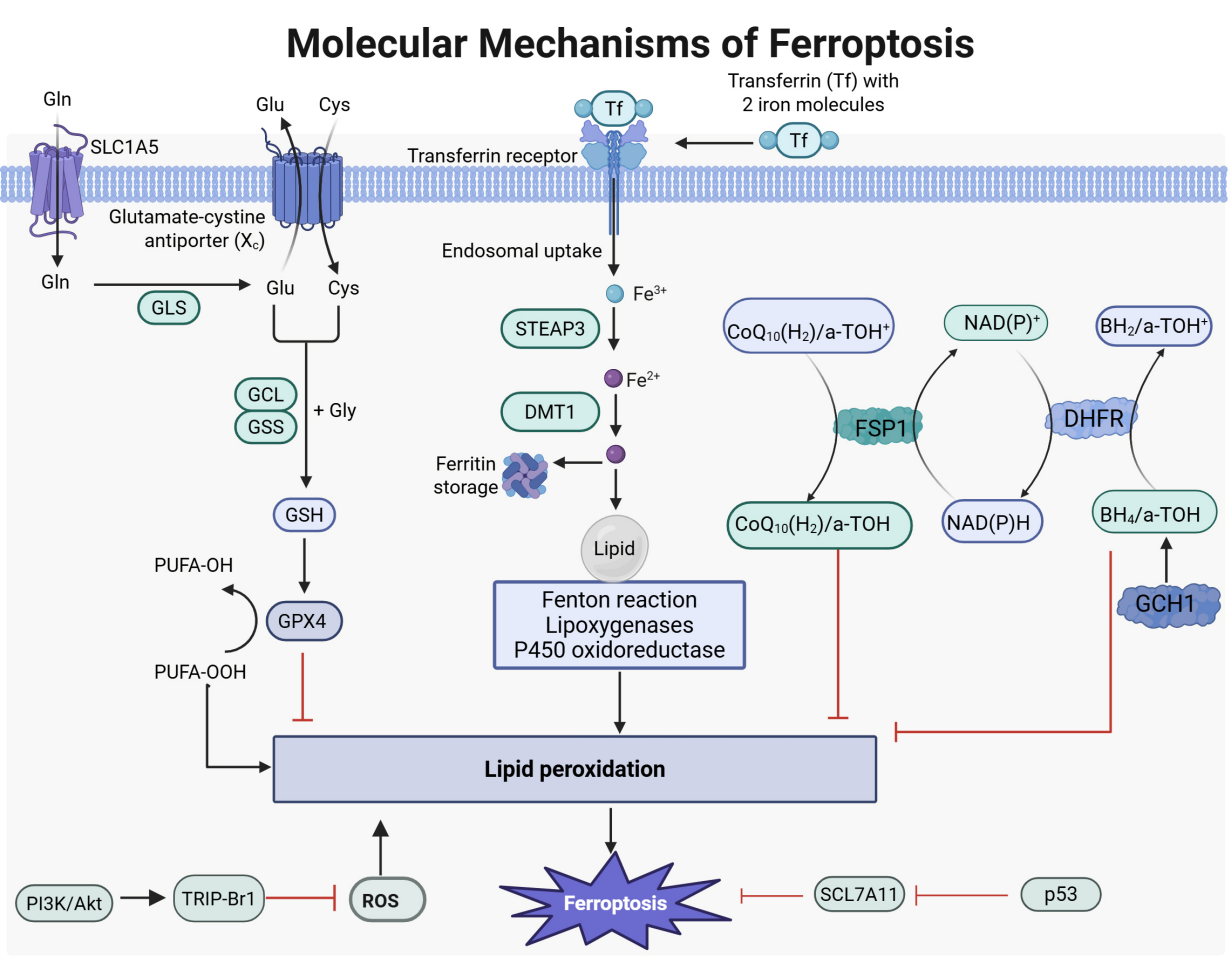
**Molecular mechanisms of ferroptosis**. Fe^3+^ binds to transferrin and is transported into the cell via transferrin (Tf) receptor. Inside, it undergoes reduction and is released into the cytosolic labile iron pool (LIP), where excess iron is stored. The intracellular LIP mainly exists as Fe^2+^. Due to Fe^2+^’s instability and high reactivity, it can generate hydroxyl radicals through the Fenton reaction. These radicals may react with polyunsaturated fatty acids in cellular membranes, leading to significant lipid reactive oxygen species (ROS) production that can cause cell death. Under physiological conditions, cystine/glutamate antiporter system (system X_C_) consists of SLC7A11 and solute carrier family 3 member 2 (SLC3A2), which transport cystine into cells. Cystine is reduced to cysteine for glutathione (GSH) synthesis—the primary intracellular antioxidant. GSH acts as a cofactor for glutathione peroxidase 4 (GPX4), facilitating its conversion from reduced GSH to oxidized GSH while reducing lipid peroxides; this helps alleviate oxidative stress injury. The interaction between the cysteine/GSH/GPX4 axis, nicotinamide adenine dinucleotide phosphate hydrogen or nicotinamide adenine dinucleotide (NAD(P)H)/ferroptosis suppressor protein 1 (FSP1)/coenzyme Q10 (CoQ10) system, and GTP cyclohydrolase 1 (GCH1)/tetrahydrobiopterin (BH4)/dihydrofolatereductase (DHFR) system is crucial in inhibiting ferroptosis. GLS, glutamine synthetase; GCL, glutamate-cysteine ligase; GSS, glutathione synthetase; STEAP3, six transmembrane epithelial antigen of prostate 3; DMT1, divalent metal transporter 1; DHFR, dihydrofolate reductase. Images created with BioRender.com.

Ferroptosis is regulated by various cellular metabolic pathways, including REDOX homeostasis, iron metabolism, mitochondrial function, and the metabolism of amino acids, lipids, and carbohydrates. Key processes affecting susceptibility to ferroptosis include the sulfhydryl-dependent REDOX system and the mevalonate pathway. Conversely, the cysteine/glutathione (GSH)/GPX4 axis, nicotinamide adenine dinucleotide phosphate hydrogen or nicotinamide adenine dinucleotide (NAD(P)H)/ferroptosis suppressor protein 1 (FSP1)/coenzyme Q10 (CoQ10) system, and guanosine triphosphate (GTP) cyclohydrolase 1 (GCH1)/tetrahydrobiopterin (BH4)/dihydrofolatereductase (DHFR) system inhibit ferroptosis. Transcription factors such as p53, NF-E2-related factor 2 (Nrf2), activating transcription factor 3 (ATF3), activating transcription factor 4 (ATF4), Yes-associated protein 1 (YAP1), hypoxia inducible factor 1 subunit alpha (HIF1α); endothelial PAS domain-containing protein 1 (EPAS1), BTB domain and CNC homolog 1 (BACH1), TcelltranscriptionfactorEB (TFEB), jun B proto-oncogene (JUNB), HIC ZBTB transcriptional repressor 1 (HIC1), and hepatocyte nuclear factor 4 alpha (HNF4α) regulate ferroptosis through transcriptional and non-transcriptional mechanisms [16]. Ferroptosis can be triggered by small molecules that inhibit GSH biosynthesis or GPX4 activity. Inducers are primarily classified into inhibitors of system Xc- and GPX4. System Xc- is a cystine/glutamate antiporter found in the phospholipid bilayer; it consists of solute carrier family 7 member 11 (SLC7A11) and regulatory subunit solute carrier family 3 member 2 (SLC3A2). This transporter is essential for maintaining intracellular GSH levels. Inhibition of system Xc- reduces cystine transport into cells—leading to lower intracellular GSH levels and decreased GPX4 activity [17]. Fig. 1 shows a diagram of the molecular mechanism of ferroptosis.

### 2.2 The Role of Ferroptosis in AS

AS is a chronic inflammatory vascular disease marked by abnormal lipid metabolism and endothelial dysfunction, with iron metabolism playing a crucial role in its development [12]. Endothelial dysfunction is the initial event in AS, and iron overload can impair endothelial function by increasing ROS production. Excessive ROS release triggers macrophage inflammation and alters lipoproteins, both of which are crucial in AS pathogenesis [18]. ROS can interact with polyunsaturated fatty acids (PUFAs) present in lipid (L) membranes, leading to the formation of lipid free radicals (L•). This interaction may subsequently induce lipid peroxidation, resulting in the generation of L-ROS [19]. Ferroptosis features include impaired clearance of lipid peroxides, redox-active iron presence, and membrane polyunsaturated fatty acid phospholipid (PUFA-PL) peroxidation [20]. Lipid peroxidation involves hydrogen atom loss from lipids due to free radicals or lipid peroxidases, leading to oxidation and fragmentation of lipid carbon chains. This process generates cytotoxic substances like lipid hydroperoxides that ultimately cause cell death. The Fenton reaction produces significant ROS that interact with PUFA and phosphatidylethanolamine (PE), promoting further lipid peroxidation. This cascade results in toxic compounds such as 4-hydroxynonenal (4-HNE) and malondialdehyde (MDA), contributing to ferroptosis [5]. Studies have identified key indicators for AS formation related to iron metabolic dysfunction—including levels of iron, GSH, GPX4, FPN, and SLC7A11 (xCT). Recent studies indicate that AS pathogenesis features ferroptosis characteristics in plaque cells, such as severe iron accumulation, reduced GPX4 levels, and increased ROS [21].

Some macromolecules, drugs, herbs, and food extracts may inhibit atherosclerosis by preventing ferroptosis in plaque cells. Gao *et al*. [22] demonstrated that Garlic reduces genes associated with ferroptosis in AS, highlighting its therapeutic potential. Wang *et al*. [23] reported that ecdysteroid alleviates AS by inhibiting NCF2 and suppressing phosphatidylinositol 3-kinase (PI3K)/protein kinase B (Akt)/Nrf2-mediated ferroptosis. Zang *et al*. [24] found that 2-Acetamidophenol (2-AAP) inhibits AS progression by reducing hyperlipidemia and attenuating the ferroptosis pathway, suggesting its therapeutic benefits for AS through these mechanisms. Zhao *et al*. [25] showed that Panax notoginseng saponins (PNS), an extract from plants, promotes NrF2-mediated inhibition of ferroptosis via reduced USP2-mediated deubiquitination of kelch-like ECH-associated protein 1 (KEAP1), thereby alleviating AS. Zhang *et al*. [26] indicated that Qing-Xin-Jie-Yu Granule inhibits ferroptosis and stabilizes plaques through modulation of GPX4/xCT signaling pathways. Puylaert *et al*. [27] revealed erythrophage-induced ferroptosis is linked to increased heme oxygenase-1 and ferritin levels; this effect can be blocked by UAMC-3203, identified as a third-generation inhibitor of ferroptosis. Meng *et al*. [28] established heme oxygenase 1 (HMOX1) upregulation enhances ferroptosis in diabetic AS development, indicating it may be a promising target for therapy or drug development in diabetes-related vascular conditions. Shi *et al*. [29] demonstrated that MaiJiTong particles reduce AS by activating signal transducer and activator of transcription 6 (STAT6), which inhibits DMT1 and suppressor of cytokine signaling 1 (SOCS1)/protein 53 (p53) pathways in low density Lipoprotein receptor (LDLR) (-/-) mice. This indicates that STAT6 is crucial for alleviating AS through ferroptosis inhibition, making it a promising therapeutic target. Thus, Maijitong (MJT) may offer an innovative treatment option for managing AS.

## 3. ECs Ferroptosis in AS 

### 3.1 The Molecular Mechanisms of Ferroptosis in ECs

The pathogenesis of AS is complex, involving various cell types such as primarily ECs and macrophages. ECs line blood vessels and are exposed to endogenous hazard signals and circulating metabolites [30]. The endothelium acts as a barrier between the bloodstream and vessel wall, regulating substance exchange among the lumen, vessel wall, and surrounding tissues while maintaining vascular homeostasis. Endothelial dysfunction or cell death can disrupt the vascular barrier, impair contraction and relaxation mechanisms, trigger inflammatory responses, and lead to thrombosis—all linked to AS progression. Oxidative stress from excessive ROS production significantly contributes to endothelial cell death. Evidence suggests that ROS can induce EC death via ferroptosis [31]. Ferroptosis has also been identified as a mode of ox-LDL-induced ECs death. Characteristics of endothelial ferroptosis can be evaluated using inhibitors like ferrostatin-1 or molecular markers such as iron content, GPX4 levels, SLC7A11, and Xc antiporter activity [30]. However, the exact molecular mechanisms governing ferroptosis in ECs remain not fully understood.

Some studies show that promoting ferroptosis in ECs can worsen AS. For instance, interleukin-17 (IL-17) is a proinflammatory cytokine that plays a role in chronic inflammation associated with allergies, cancers, and autoimmune diseases like rheumatoid arthritis, systemic lupus erythematosus, multiple sclerosis, and psoriasis [32]. Gu *et al*. [33] showed interleukin-17d promotes endothelial cell ferroptosis through CD93 (also known as complement protein 1 q subcomponent receptor C1qR1 or C1qRp)/miR-181a-5p/SLC7A11 pathways, accelerating AS development. Fang *et al*. [34] demonstrated that sequestosome 1 (SQSTM1) upregulation-induced iron overload triggers nicotine-exacerbated endothelial ferroptosis in AS.

Some studies have demonstrated that the promotion of ferroptosis in ECs can enhance AS. For example, the study by Bai *et al*. [35] showed that the ferroptosis inhibitor ferrostatin-1 (Fer-1) significantly increased levels of key markers SLC7A11 and GPX4, while downregulating adhesion molecules and upregulating endothelial nitric oxide synthase (eNOS) expression. Inhibition of ferroptosis alleviates AS by reducing lipid peroxidation and endothelial dysfunction in aortic endothelial cell (AEC). Su *et al*. [36] reported that radiation-induced endothelial ferroptosis accelerates AS progression, with DDHD2 identified as a potential regulatory protein involved via the Nrf2/GPX4 pathway. Rong *et al*. [37] found that Hydroxysafflor yellow A inhibits endothelial cell ferroptosis in diabetic atherosclerotic mice through modulation of miR-429/SLC7A11. Wang *et al*. [38] established that decreased mRNA levels of sterol regulatory element-binding protein (SREBP-1) in peripheral blood are an independent risk factor for stable coronary artery disease (CAD), indicating that SREBP-1-mediated lipid biosynthesis can mitigate endothelial injury by counteracting ferroptosis. Du *et al*. [39] demonstrated that C1q/TNF-Related Protein 13 (CTRP13) enhances mitochondrial oxidative stress response, inhibits endothelial cell ferroptosis, and improves function via the GCH1/BH4 signaling pathway, thus hindering AS progression. Zhang *et al*. [40] found Qixian granules inhibit vascular endothelial cell ferroptosis by regulating Transient Receptor Potential Mucolipin 1 (TRPML1) within lysosomes, preventing postmenopausal AS. He *et al*. [41] reported that elevated circulating LncRNA NORAD promotes endothelial cell growth and mitigates ferroptosis by regulating the miR-106a/cyclin D1 (CCND1) axis in CAD patients. Tan *et al*. [42] found that atorvastatin alleviates endothelial cell damage in AS by inhibiting acyl-CoA synthetase long chain family member 4 (ACSL4)-mediated ferroptosis. Hu *et al*. [43] revealed that adrenomedullin is transcriptionally regulated by the vitamin D receptor, which helps alleviate AS in mice through Adenosine 5^′^-monophosphate (AMP)-activated protein kinase (AMPK)-mediated endothelial ferroptosis inhibition. Zaitoun *et al*. [44] showed that plasma fibronectin (FN) prevents acrolein-induced ferroptosis in ECs by reducing lipid peroxidation and inflammation while reversing biomarkers associated with ferroptosis, mediated through the AMPK/Nrf2 signaling pathway, which upregulates GPX4 and SLC7A11 expression—key regulators of ferroptosis. Collectively, these studies elucidate various molecular mechanisms underlying endothelial cell ferroptosis in AS; however, further exploration into comprehensive molecular pathways remains necessary.

### 3.2 Therapeutic Targets for Ferroptosis in ECs

ECs injury and inflammation are key factors in the onset and progression of AS. RCD of ECs contributes to endothelial dysfunction, leading to local denudation and thrombosis. This process results in lipid and fibrous component deposition within large and medium-sized arteries, facilitating AS development. Therefore, effectively regulating the RCD of ECs is crucial for preventing and treating AS [45]. Recent studies have identified ferroptosis in ECs—driven by iron-dependent lipid peroxidation and ROS accumulation—as a pathogenic mechanism in AS development. This highlights ferroptosis’s significant role in promoting lipid peroxidation, which causes endothelial injury. Thus, targeting ferroptosis in ECs has emerged as a promising therapeutic strategy for addressing AS [46].

Zhu *et al*. [46] reported elevated nuclear receptor coactivator 4 (NCOA4) expression in ApoE mice and ECs, significantly associated with AS. The upregulation of NCOA4 promotes ferroptosis, with lectin-like oxidized low-density lipoprotein receptor-1 (LOX-1) identified as a key upstream target influencing its function. This pathway is linked to cyclic GMP-AMP synthase (cGAS)-stimulator of interferon genes (STING) signaling activation, enhancing NCOA4 expression. These findings suggest that the “Gualou-Xiebai” herbal pair, targeting LOX-1—an upstream molecule of NCOA4—may be a potential therapeutic strategy for AS. He *et al*. [47] demonstrated that Gsα protects against endothelial ferroptosis and may be a therapeutic target for atherosclerosis and ischemic heart disease. Gsα regulates NRF2 expression and inhibits ferroptosis via cyclic adenosine monophosphate (cAMP)/exchange protein activated by cAMP (Epac)/CCCTC-binding factor (CTCF)-mediated transcription. Wang *et al*. [48] demonstrated that 6-Gingerol inhibits ferroptosis in atherosclerotic ECs by activating the nuclear factor erythroid 2-related factor 2 (NRF2)/heme oxygenase-1 (HO-1) pathway, suggesting that targeting endothelial ferroptosis may effectively treat AS. Zeng *et al*. [49] found that Itchy E3 ubiquitin ligase (ITCH) interacts with ferritin light chain (FTL) protein and modulates its stability through the ubiquitin-proteasome system, leading to ox-LDL-induced ferroptosis and subsequent dysfunction of ECs. Targeting components within the ITCH-FTL pathway shows promise as an innovative therapeutic strategy against AS. The findings of Chen *et al*. [50] confirmed that oxidized phospholipid 1-palmitoyl-2-glutaryl-sn-glycero-3-phosphocholine (PGPC) promotes ferroptosis in ECs via fatty acid binding protein 3 (FABP3), impairing endothelial function. This study offers new insights into AS mechanisms and identifies potential therapeutic targets. Zhu *et al*. [51] reported that the Gualou-Xiebai herbal pair improved AS in high-fat diet (HFD)-induced ApoE (-/-) mice and mitigated ox-LDL damage to human umbilical vein endothelial cells (HUVECs) by regulating NrF2-mediated ferroptosis. Wang *et al*.’s experiments [52] revealed that Icariin alleviates ferroptosis-related AS by promoting autophagy in ox-LDL-induced vascular endothelial cell injury within an atherosclerotic mouse model. Xiang *et al*.’s study [53] unveiled that the metabolite Neu5Ac may promote SLC3A2-associated endothelial ferroptosis, leading to EC damage and AS plaque progression; this provides fresh insights into the Neu5Ac-ferroptosis pathway’s role in AS development and suggests pharmacological inhibition of ferroptosis as an innovative early-onset AS therapy strategy. In addition, an early clinical trial has demonstrated that deferoxamine, an iron chelator, can enhance endothelial function in patients with coronary artery disease through nitric oxide-mediated endothelium-dependent vasodilation [54].

## 4. Macrophages Ferroptosis in AS

### 4.1 The Molecular Mechanisms of Ferroptosis in Macrophages

Atherosclerosis is characterized by massive macrophage infiltration, and macrophages in plaques can undergo ferroptosis [55]. In the process of ferroptosis, ferric iron is reduced to divalent iron, resulting in the release of ROS. This cascade induces and promotes the formation of lipid peroxides, further exacerbating oxidative stress damage within cells and consequently influencing the progression of AS. Macrophages are key mediators in AS progression and iron metabolism; thus, modulating their iron metabolism may be an important strategy for stabilizing plaques and inhibiting AS progression [56]. Some cytokines, primarily from macrophages, can induce or inhibit ferroptosis in various ways, including interleukin (IL)-6, tumour necrosis factor alpha (TNF-α), IL-1β, and inducible nitric oxide synthase (iNOS) [57].

Elucidating the molecular mechanisms of macrophage ferroptosis is crucial for understanding AS. Bao *et al*. [58] showed that cigarette tar induces macrophage ferroptosis in AS via the hepcidin/FPN/SLC7A11 signaling pathway. An nuclear factor kappa B (NF-κB) inhibitor (BAY11-7082) counteracted tar’s effects on this axis, inhibiting macrophage ferroptosis. Yang *et al*. [59] reported that AMPK signaling is a key regulator of metabolism and significantly influences ferroptosis; its inhibition reduces AS and foam cell formation by modulating lipid metabolism through AMPK activation. Lin *et al*. [60] found that ricetin inhibits macrophage ferroptosis by activating the NRF2 pathway, alleviating AS. Hu *et al*. [61] revealed that P2Y12 inhibition decreases autocrine hepcidin production by preventing NF-κB p65 phosphorylation in macrophages, helping to avoid intracellular iron retention and subsequent AS development. Tao *et al*. [62] demonstrated that melatonin (MLT) suppresses Lp-PLA2 expression and slows AS progression by inhibiting macrophage ferroptosis while partially activating the NRF2 pathway. Emerging evidence highlights IL-37 as a protective cytokine that activates the Nrf2 pathway in murine models, inhibiting macrophage ferroptosis and slowing AS progression [63]. Luo *et al*. [64] reported that micelliolide (MCL) alleviates AS by targeting KEAP1/NRF2 interactions to inhibit macrophage ferroptosis.

Liu *et al*. [65] found that FUT8-regulated Unc5b fucosylation reduces macrophage migration and accelerates AS via the ferroptosis pathway, providing new perspectives on its pathophysiology. Pei *et al*. [66] demonstrated that miR-214-3p enhances ox-LDL-induced macrophage ferroptosis and inflammation through GPX4 modulation. The findings of Guo *et al*. [67] show that high levels of heme oxygenase-1 promote ferroptosis in macrophage-derived foam cells, leading to plaque instability. Under hypoxic conditions, elevated Hmox1 expression negatively impacts the viability of myeloid-derived suppressor cells (MDFCs) and plaque stability, offering insights for managing acute cardiovascular events. Together, these findings offer valuable insights into the mechanisms of macrophage ferroptosis in AS.

### 4.2 Therapeutic Targets for Ferroptosis in Macrophages

Advanced AS is the pathological basis for acute cardiovascular events and significantly raises the risk of recurrence, even with modern therapies. The death of foam-like macrophages is critical to plaque progression. RNA sequencing indicates that iron accumulation in advanced AS promotes ferroptosis in foamy macrophages [68]. Therefore, targeting macrophage ferroptosis is essential for developing therapeutic strategies against AS [69].

Targeting macrophage ferroptosis offers promising avenues for novel AS prevention and treatment strategies. Yang *et al*. [70] showed that pitavastatin and resveratrol nanocomposites protected against hyperhomocysteinemia-induced AS by blocking ferroptosis-related lipid deposition. MCL is an active metabolite of parthenolide. Luo *et al*. [71] demonstrated that MCL inhibits macrophage ferroptosis via the NRF2 pathway to mitigate AS. Chen *et al*. [72] reported that spermine delivered by ZIF90 nanoparticles alleviated AS by specifically targeting macrophage ferroptosis within plaques. Jin *et al*. [73] illustrated that polylactic-co-glycolic acid nanoparticles loaded with astaxanthin inhibited macrophage ferroptosis via the NRF2/SLC7A11/GPX4 signaling pathway, alleviating symptoms associated with AS.

**Fig. 2.**
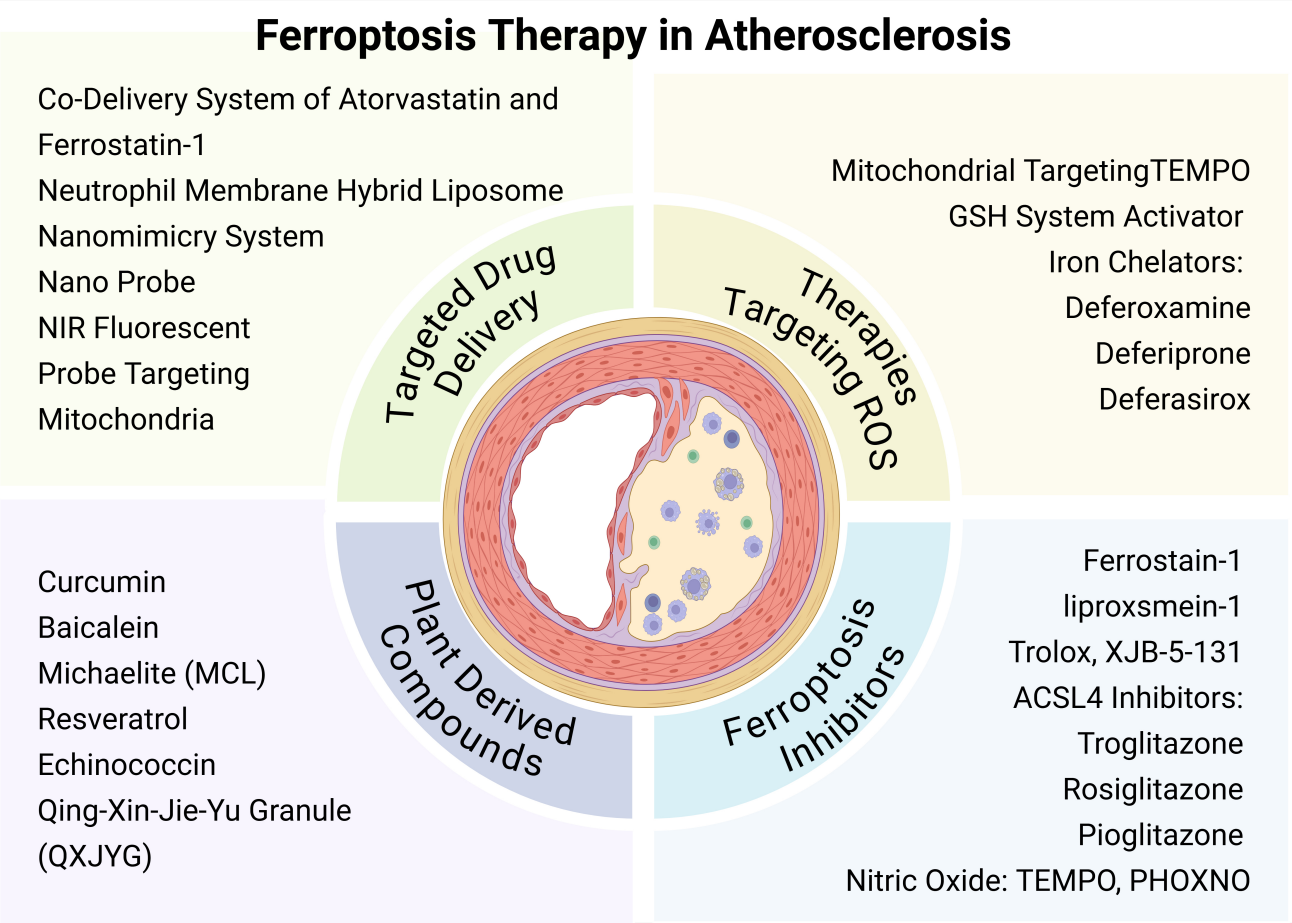
**Ferroptosis therapy in atherosclerosis (AS)**. Targeted ferroptosis therapy for AS includes four main aspects: strategies to reduce reactive oxygen species (ROS), ferroptosis inhibitors, plant-derived compounds, and targeted drug delivery systems. TEMPO, 2,2,6,6-Tetramethylpiperidine-1-oxyl; PHOXNO, 2-(2-Phenyl-2-oxoethyl)-2,5,5-trimethylpyrrolidine-N-oxyl. Images created with BioRender.com.

Current studies are investigating therapeutic strategies to mitigate the oxidative effects of ROS, particularly through the reduction of GSH. GSH, a tripeptide synthesized mainly in the heart and liver, plays crucial roles in cellular homeostasis and acts primarily as an antioxidant. In addition to GSH system activators, iron chelators may provide therapeutic benefits for patients with excessive free radical production due to iron toxicity. The U.S. food and drug administration (FDA) has approved three major iron chelators for clinical use: deferoxamine, deferiprone, and deferasirox [74]. Alongside ferroptosis inhibitors, several novel therapies targeting ferroptosis have emerged. These include new applications for existing drugs, plant-derived compounds, and targeted drug delivery systems [75]. Feng *et al*. [76] reported a hybrid exosome/liposome system that co-delivers atorvastatin and Ferrostatin-1 to inhibit both ferroptosis and inflammation while promoting exocytosis and macrophage reprogramming in AS treatment. Beyond inhibiting ferroptosis, Ferrostatin-1 enhances macrophage exocytosis potentially via MAPK pathway activation. This study presents an integrated approach for treating AS, proposing various drug combinations that highlight Ferrostatin-1’s potential as an adjuvant anti-AS agent while offering promising avenues for advanced AS therapy. Li *et al*. [77] developed a hybrid neutrophil membrane liposome nanomimicry system (Ptdser-NM-Lipo/Fer-1) for targeted delivery of Fer-1 to atherosclerotic plaques. This system features PtdSer-modified liposomes encapsulating Fer-1, surrounded by a neutrophil shell. Upon reaching the plaques, Fer-1 is released to eliminate ROS and improve the inflammatory microenvironment, highlighting its potential as a novel therapeutic approach for AS. Gu *et al*. [78] utilized macrophage membrane-engineered nanoprobes to visualize ferroptosis in atherosclerotic plaques, providing insights into ferroptosis’s role in diagnosis and treatment strategies for AS. Huang *et al*. [79] introduced an innovative mitochondrial-targeted near-infrared (NIR) fluorescent probe for viscosity detection, featuring various electron donor groups; notably, Mito-Vis-4 has an extensive Stokes shift (200 nm) and mitochondrial targeting capabilities, making it suitable for imaging viscosity changes during ferroptosis and serving as a promising non-invasive monitoring tool. Fig. 2 provides a comprehensive overview of targeted ferroptosis therapies for AS. Collectively, these findings may inspire new ideas and identify potential targets for preventing and treating AS. Significant progress has been made in researching drugs that target ferroptosis, yet challenges remain regarding drug safety, selectivity, and delivery systems. With advancements in science and technology—alongside precision medicine and innovative delivery methods—targeted ferroptosis therapy is set to play a vital role in clinical practice.

## 5. Iron Supplementation and Antioxidant Clinical Studies

In addition, clinical studies on iron supplementation and AS indicate that long-term high-dose oral treatment for iron deficiency anemia (IDA) can lead to tissue iron overload and oxidative stress, initiating the atherosclerotic process [80]. Furthermore, statins improve the high-density lipoprotein (HDL) to LDL ratio and lower ferritin levels through non-interacting mechanisms, suggesting a link between the clinical benefits of statins and maintaining physiological iron levels. This implies that reducing iron could be a safe, low-cost alternative to statins [81]. Non-transferrin bound iron in plasma from dialysis patients after iron gluconate infusion can catalyze hydroxyl radical formation, potentially causing cell damage and promoting atherosclerosis [82]. Excess body iron correlates with elevated circulating oxysterol levels based on serum ferritin assessments in humans [83]. Other studies show that crocin supplementation significantly improves inflammation, oxidative stress status, and leptin levels in CAD patients [84]. Antioxidant carotenoids help reduce oxidative lipid products and inflammation systemically, thereby lessening their role in forming atherosclerotic plaques. Lutein, zeaxanthin, and meso-zeaxanthin supplements have been shown to decrease inflammatory cytokines and markers of oxidative cardiovascular processes in humans [85]. Orlistat is known as a reversible inhibitor of pancreatic and gastric lipases with antioxidant properties; clinical studies suggest it plays an important role in endothelial dysfunction and the processes of atherosclerosis via oxosterol modulation [86].

## 6. Conclusions and Future Perspectives

Atherosclerosis is a CVD that significantly threatens human health. The role of ferroptosis in the initiation and progression of atherosclerosis is increasingly recognized. Ferroptosis involves lipid peroxidation, iron dysregulation, and antioxidant pathways. Evidence indicates that ferroptosis in ECs and macrophages is closely linked to atherosclerosis. Iron, an essential mineral for macrophage function under normal conditions, can lead to overload and promote the progression of atherosclerosis through ferroptosis. The ferroptosis of ECs and macrophages contributes to the instability of atherosclerotic plaques. Thus, understanding and regulating the molecular mechanisms behind ECs and macrophages ferroptosis may provide important strategies for stabilizing plaques and inhibiting disease progression. Targeting this process could offer promising therapeutic avenues for atherosclerosis. Recent studies have advanced our understanding of the molecular mechanisms involved in ferroptosis within ECs and macrophages related to atherosclerosis, yielding positive results in targeted treatments. However, despite significant progress in basic research, there is still a considerable gap to successful clinical translation. Additionally, studies on clinical drugs targeting ferroptosis remain limited. Moving forward, more clinical trials are needed to explore effective treatment strategies. Comprehensive research efforts are crucial to unravel these complexities; achieving deeper insights will enhance targeted therapeutic interventions effectively.
